# A drug repurposing screen identifies antiviral compounds against Puumala Orthohantavirus

**DOI:** 10.1038/s41598-026-57843-1

**Published:** 2026-06-25

**Authors:** Wanda Christ, Bartlomiej Porebski, Oscar Fernandez-Capetillo, Jonas Klingström

**Affiliations:** 1 Division of Virology and Immunology, Department of Microbiology, Tumor and Cell Biology, Karolinska Institutet, Stockholm, Sweden; 2 Division of Genome Biology, Science for Life Laboratory, Department of Medical Biochemistry and Biophysics, Karolinska Institutet, Stockholm, Sweden; 3 Chemical Biology Consortium Sweden, Science for Life Laboratory, Department of Medical Biochemistry and Biophysics, Karolinska Institutet, Stockholm, Sweden; 4 Genomic Instability Group, Spanish National Cancer Research Centre (CNIO), Madrid, Spain; 5 Networking Research Center on Neurodegenerative Diseases (CIBER-NED), Instituto de Salud Carlos III, Madrid, Spain; 6 Division of Molecular Medicine and Virology, Department of Biomedical and Clinical Sciences, Linköping University, Linköping, Sweden

**Keywords:** Orthohantavirus, Hantavirus, Puumala virus, Drug repurposing screen, Antiviral, Endothelial cells, Antibiotics, Computational biology and bioinformatics, Diseases, Drug discovery, Microbiology

## Abstract

Hantaviruses are zoonotic negative-sense RNA viruses that cause haemorrhagic fever with renal syndrome (HFRS) and hantavirus pulmonary syndrome (HPS), yet no approved antiviral therapies are available. To identify host-directed modulators of hantavirus infection we performed a drug repurposing screen using live Puumala virus (PUUV). We identified and validated 70 drugs with antiviral activity in A549 cells and primary human endothelial cells. Functional clustering confirmed the known infection-inhibitory effect of several groups of compounds, including inhibitors of heat shock proteins, mTOR pathway and nucleotide synthesis. Our screen also identified compounds yet unexplored as antivirals against Hantaviruses, such as certain antibiotics. Our dataset provides a systematic map of host pathways influencing PUUV infection and highlights candidate compounds and cellular processes that can modulate this process.

**Supplementary Information:**

The online version contains supplementary material available at 10.1038/s41598-026-57843-1.

## Introduction

Orthohantaviruses (hereafter referred to as hantaviruses) are zoonotic, negative-sense RNA viruses of the *Hantaviridae* family carried by rodents and other small animals^1^. Transmission to humans occurs through inhalation of aerosols contaminated with rodent droppings, saliva, or urine. Human-to-human transmissions are rare but have been reported^2^. Of the > 60 hantaviruses identified to date, several rodent-borne hantaviruses are associated with two human diseases: hantavirus pulmonary syndrome (HPS) or haemorrhagic fever with renal syndrome (HFRS)^3^. Annual incidence and case fatality vary by viral species and geographic region^2^. HPS is endemic to South and North America and is associated with case fatality rates of 30 to 40%^2,4–7^. In contrast, HFRS occurs predominantly in China, South Korea and parts of Europe, with case fatality rates normally below or around 1% but reaching up to 10% for certain hantaviruses^2^. Puumala virus (PUUV) is the most common hantavirus in Europe, where it causes HFRS with < 1% case fatality. While HFRS typically affects the kidneys and HPS mainly targets the lungs, symptoms of both diseases partly overlap, starting with flu-like symptoms with high fever, headache, myalgia, nausea, and gastrointestinal symptoms, and both can eventually lead to sudden organ failure and shock^2^.

To date, no EMA- or FDA-approved vaccine or hantavirus-specific treatment is available, and the treatment of hantavirus disease is limited to symptom management^2,8^. Over the past decades, hypothesis-driven studies have proposed several antiviral strategies that showed promise in vitro or in animal models but have failed to demonstrate significant benefits in clinical settings (reviewed in^9,10^. Reported drug discovery efforts have relied on pseudotyped viruses^11,12^ or focused on specific facets of infection^13,14^. While informative, such approaches do not account for possible host dependencies in the context of productive live-virus infection and replication. To address these limitations, we conducted a microscopy-based phenotypic high-throughput drug repurposing screening to identify host-directed modulators of PUUV infection.

## Results

### A high-throughput screening platform to identify modulators of PUUV-infected cells

**Fig. 1:**
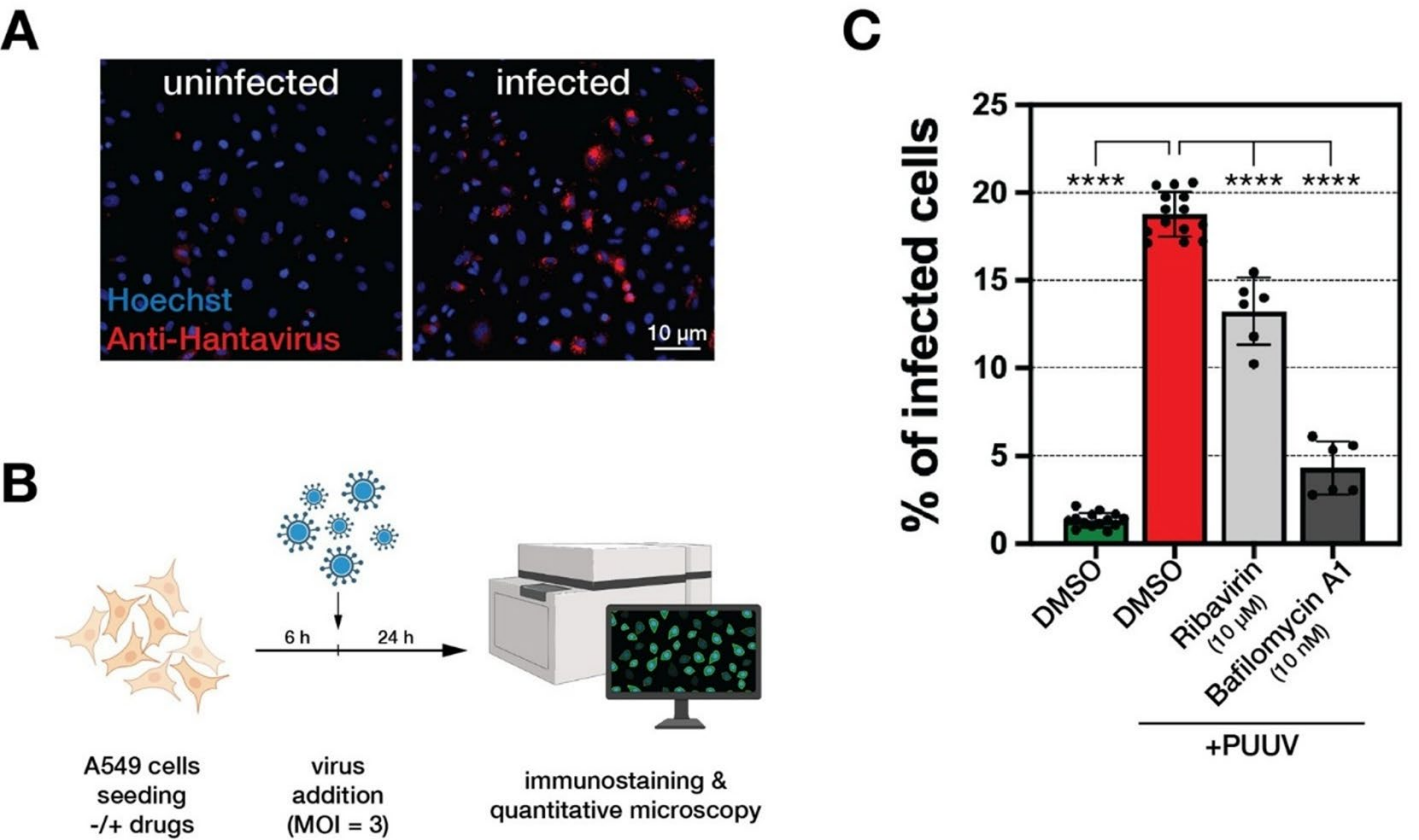
Screening assay development. **A.** Representative microscopic images of uninfected (left) or PUUV-infected (right) A549 cells. Viral antigen was detected using polyclonal antibody from convalescent sera (red), and nuclei were counterstained with Hoechst 33342 (blue). Scale bar, 10 μm. **B.** Schematic of the screening assay. A549 cells were seeded together with small molecule compounds and incubated for 6 h. PUUV was then added at MOI = 3, and 24 h post infection cells were fixed and subjected to immunostaining and quantitative microscopy. **C.** Quantification of PUUV infection in A549 cells following treatment with vehicle (DMSO), 10 µM Ribavirin, or 10 nM Bafilomycin A1. Infection rate was calculated as the percentage of virus-positive cells. Dots represent individual wells, each with 2000–2500 cells. Bars indicate mean ± SD. Statistical analysis was done using ANOVA with Dunnett’s test for multiple comparisons; ****, *p* < 0.0001.

To identify new antiviral compounds against hantavirus infection, we used a phenotypic assay based on PUUV infection of lung adenocarcinoma A549 cells (**Fig. ****1****A**). To maximise the performance and enable automation, we needed to streamline the assay protocol. Standard infection protocol that includes medium removal, 1 hour incubation with the virus, followed by wash, and release into virus-free medium is not compatible with high-throughput approach. Therefore, we attempted to simplify the protocol by adding the virus directly to the growth medium and omitting all the washing steps. We could observe productive infection, albeit at lower efficiency, which we then mitigated by increasing the virus dose (**Fig. ****S1****A-B**). Finally, we simplified sample preparation for microscopy without compromising staining quality (**Fig. ****S1****C**). In the final screening assay (**Fig. ****1****B**), cells were seeded together with compounds, incubated for 6 hours, followed by a direct addition of PUUV at multiplicity of infection (MOI) of 3. Twenty-four hours after the infection, cells were fixed, immunostained against viral proteins using convalescent sera, and counter-stained with Hoechst 33342 and CellTracker to mark DNA and cell body, respectively. Samples were then imaged with high-throughput high-content automated microscopy and images were analysed in Cell Profiler^15^. Infection rates were calculated as the ratio of virus signal-positive cells to total number of cells. The screening assay showed a reproducible infection rate of about 20% with low within- and between-plates variation (**Fig. ****S1****D**), providing a good screening window (Z’ = 0.7, S/*N* = 42). The assay was sensitive enough to detect both mild and strong antiviral effects of two tested control compounds, Ribavirin^16^ and Bafilomycin A1^17^(**Fig. ****1****C**).

### Drug repurposing chemical screen for modulators of PUUV infection

**Fig. 2:**
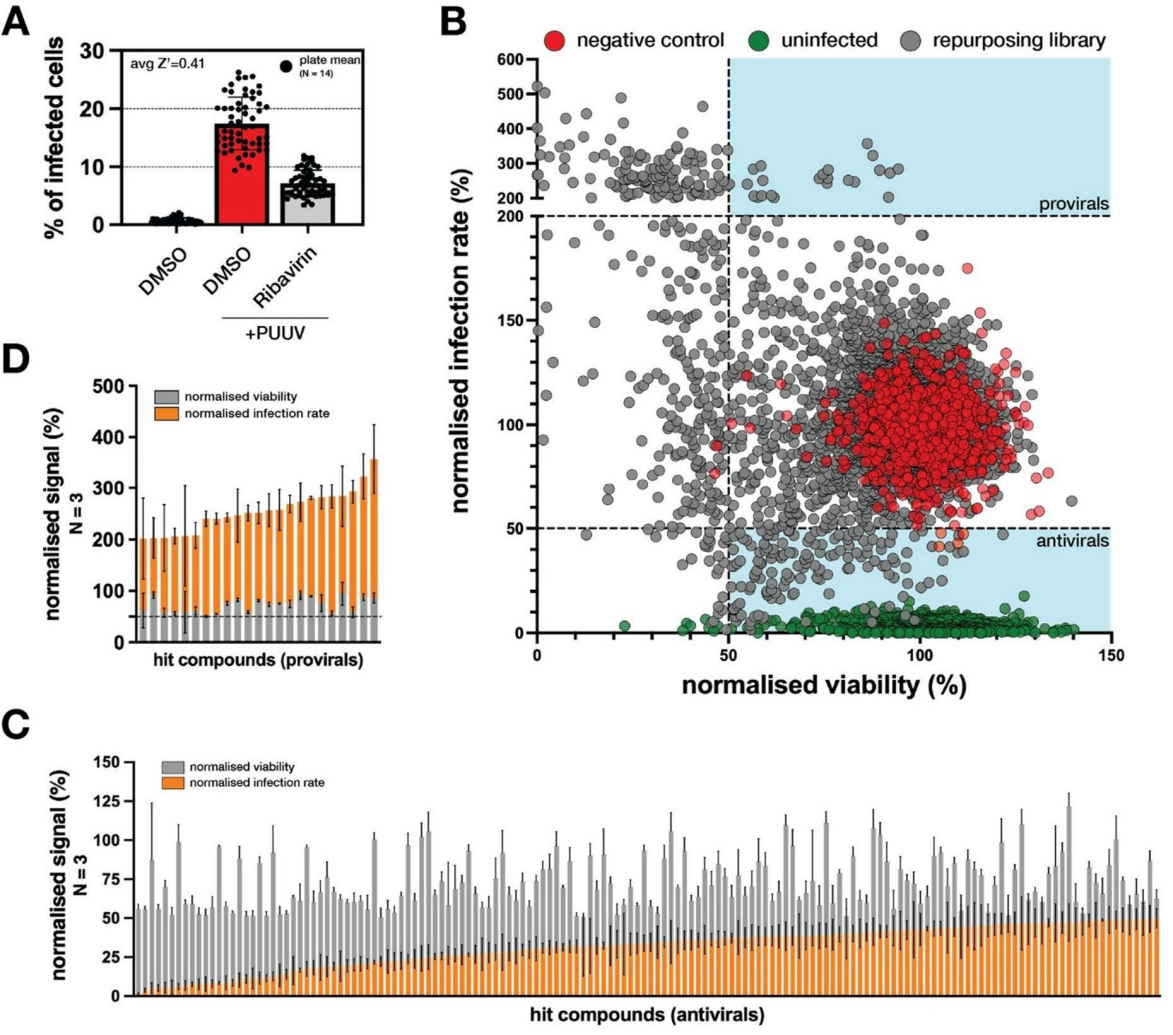
Primary screening data. **A**. Bar graph showing raw infection rate in the control samples. Each data point represents 1 screening plate (14 samples per plate); bars indicate mean ± SD. **B**. Scatter plot of the primary screening results showing normalised infection rate versus normalised viability. Normalisation was done to DMSO-treated PUUV-infected samples. Grey dots represent screened compounds, red dots indicate vehicle control, and green dots indicate uninfected controls. Dashed line denote thresholds for hit calling. Shaded areas indicate regions classified as hits. **C**. Bar graph with superimposed normalised viability (grey) and normalised infection rate (orange) for all candidate antivirals. Bars indicate mean ± SD from triplicate screening plates. **D**. Bar graph with superimposed normalised viability (grey) and normalised infection rate (orange) for all candidate provirals. Bars indicate mean ± SD from triplicate screening plates.

Next, we performed the screen using the Drug Repurposing Hub library, a curated collection of 5256 FDA-approved, clinical, and pre-clinical drugs^18^, to identify compounds that affect PUUV-infection. In the screen, A549 cells were seeded onto 384-well plates with pre-spotted library compounds (10 µM, triplicate plates) and then processed as described above. All data were normalised to the infection rate observed in vehicle (DMSO)-treated samples. In parallel, viability was calculated as the vehicle-normalised nuclei count. Data were normalised within each plate to account for plate-to-plate and batch-to-batch variation. Across the full screen, the mean infection rate was 17.7% with limited batch-to-batch variation (**Fig.** **2****A**; **Fig. ****S2****A**). The Z’ across the full screen was 0.41 (**Fig. ****S2****B**) reflecting increased biological variability at scale but remaining within an acceptable range for phenotypic screening. Low Z’ for certain plates was due to the variation in the vehicle-treated control samples (**Fig. ****S2****C**). For candidate antiviral hit calling, we selected two criteria: compounds had to decrease the infection rate at least two-fold and show maximum 50% viability decrease (**Fig.** **2****B**). One hundred fifty-one compounds passed these thresholds (**Fig.** **2****C**). Interestingly, 23 compounds led to an increase in the number of infected cells, after passing the applied thresholds (**Fig.** **2****D**).

Together, the screen demonstrated stable performance at scale and identified 151 antiviral and 23 proviral candidate compounds for subsequent validation.

### Validation of screening hits

**Fig. 3:**
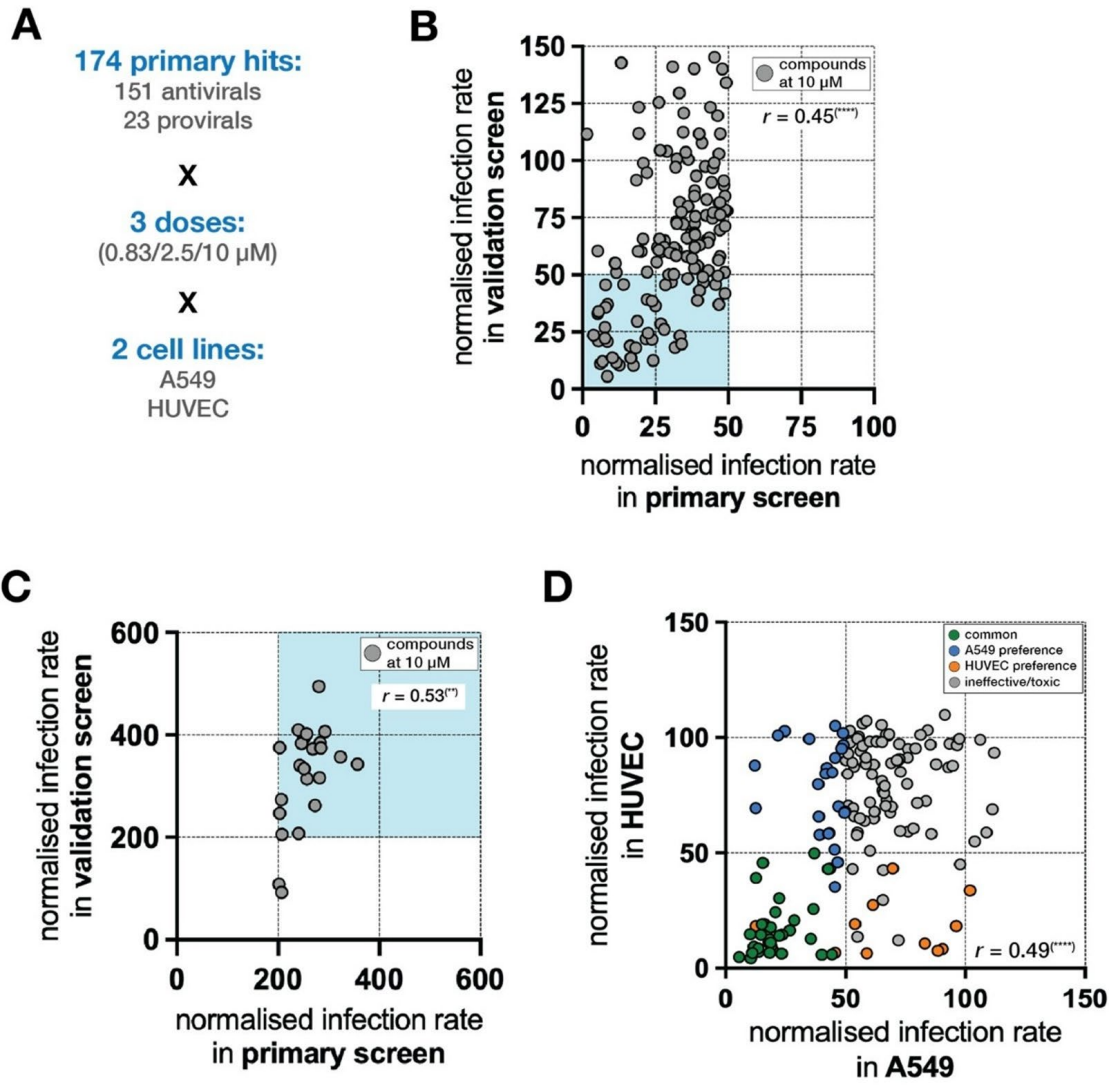
Validation of primary screening hits and cross-cell comparison. **A**. Overview of the validation strategy. All pro- and antiviral candidates were tested at 3 doses, in triplicates, in A549 cells and HUVECs. **B**. Scatter plot showing correlation between infection rate measured for antiviral compounds in the primary screen and in the validation experiment in A549 cells at 10 µM. Each dot represents one compound (mean of triplicates). Pearson correlation coefficient, *r*, is indicated. Shaded area indicates validated hits. **C**. Scatter plot showing correlation between infection rate for proviral compounds measured in the primary screen and in the validation experiment in A549 cells at 10 µM. Each dot represents one compound (mean of triplicates). Pearson correlation coefficient, *r*, is indicated. Shaded area indicates validated hits. **D**. Comparison of maximal antiviral effect between A549 cells and HUVECs. Each dot represents one compound; infection rates correspond to the concentration at which each compound produced its strongest antiviral effect. Pearson correlation coefficient, *r*, is indicated.

We next validated the hits from the primary screen. We tested each of the 151 antiviral and 23 proviral candidates at 3 different doses, including the screening dose, in 2 different cell types (**Fig.** **3****A**). As an initial check of reproducibility, we compared primary screening data with dose-matched (10 µM) validation assay data for A549 cells. We observed moderate correlation between datasets (*r* = 0.45). Most tested compounds showed antiviral effects, although the effect sizes differed between experiments (**Fig.** **3****B**). Among proviral compounds, 21 out of 23 primary hits were validated in A549 cells (**Fig.** **3****C**).

Given that hantaviruses primarily target endothelial cells, we next tested antiviral hits in primary human umbilical vein endothelial cells (HUVEC) (**Fig. ****S2****D**). We observed substantial concordance between the data obtained in the 2 tested cell systems (**Fig. ****3****D**). Majority of the compounds showed varying level of antiviral activity in either or both cell lines. Using the same thresholds as for the primary screening, we concluded that 70 of the 151 primary screening hits were validated. Twenty-five were specific to A549 cells, 11 to HUVECs, and 34 were active in both cell types.

High infection rate in HUVECs precluded investigation of proviral effects in this system (**Fig. ****S2****E**).

### Functional clustering of hit compounds

**Fig. 4:**
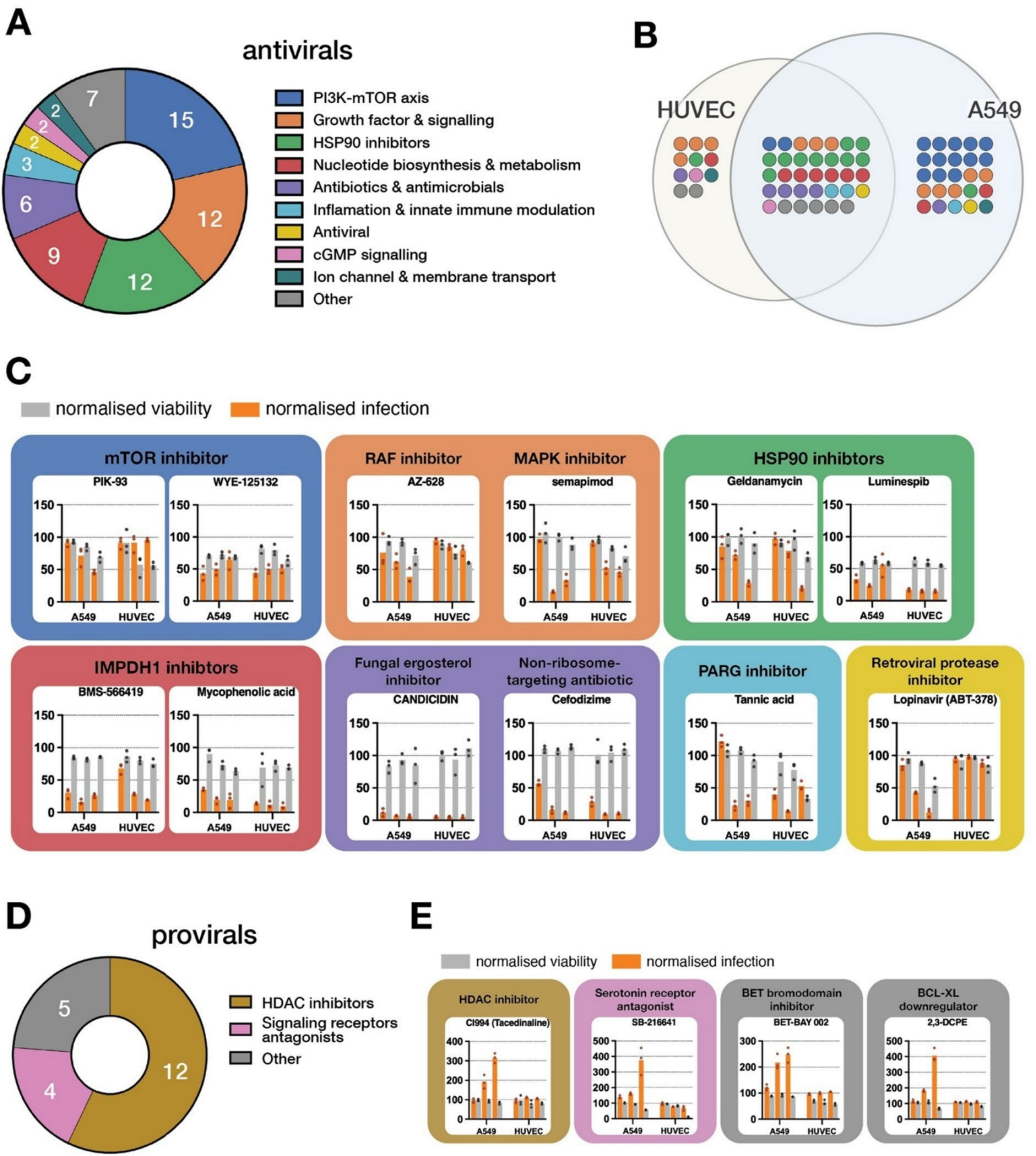
Functional classification of validated hit compounds. **A**. Functional distribution of validated antiviral compounds grouped according to annotated targets or mechanisms of action. Compounds were binned into higher-level categories based on Drug Repurposing Hub annotations and literature curation. Numbers indicate the number of compounds per class. **B**. Venn diagram showing overlap of antiviral compounds between A549 cells and HUVECs. Each dot represents one compound; colours correspond to functional classes shown in panel A. **C**. Representative dose-response (0.83, 2.5, 10 µM) profiles for selected antiviral compounds from major functional classes. Normalised infection rate (orange) and normalised viability (grey) are shown for A549 cells and HUVECs. Data for all validated antiviral compounds are provided in Supplementary Figure S3. **D**. As in A, but for candidate provirals. **E**. As in C, but for candidate provirals. Data for all validated proviral compounds are provided in Supplementary Figure S4.

One advantage of drug repurposing is the availability of prior information about the compounds, including their annotated targets and mechanisms of action. We extracted official annotations and grouped compounds into higher-level classes; missing annotations were completed by literature curation. This analysis revealed clustering of validated hits into several compound classes (**Fig. ****4****A**), including mTOR inhibitors, HSP90 chaperone inhibitors, and compounds affecting cell signalling or nucleotide synthesis. Cross-referencing functional classes with cell type-specificity showed that most compound classes were active in both A549 cells and HUVECs. An exception was mTOR inhibitors, which were particularly effective at inhibiting infection in A549 cells (**Fig. ****4****B**). Notably, several non-ribosome-targeting antibiotics were also present among the validated compounds. To illustrate the range of dose-response behaviours observed within functional classes, we show dose-response profiles for selected example compounds across the identified clusters (**Fig. ****4****C**). Dose-response curves for all validated antiviral compounds are shown in **Fig. ****S3**. Finally, functional clustering of the compounds that increased the infection rate in A549 cells revealed enrichment of epigenetics modifying compounds, particularly histone deacetylase (HDAC) inhibitors (**Fig. ****4****D**). The response profile of selected compounds in this group are shown in **Fig. ****4****E**. Dose-response curves for all validated proviral compounds are shown in **Fig. ****S4**. Taken together, the reproducibility of antiviral effects across experiments in A549 cells and HUVECs, combined with non-random clustering of hits into functional classes, supports the internal consistency of the screening results and suggests possible targets and signalling pathways for therapeutic strategies using repurposed drugs.

## Discussion

Hantaviruses, including PUUV, cause severe human diseases, yet no approved antiviral therapies are currently available and patient management remains largely supportive^2^. Although multiple targeted antiviral strategies have been explored, systematic interrogation of host dependencies during productive hantavirus infection has remained limited. Here, we addressed this gap by applying a phenotypic, live-virus screening approach to identify modulators of PUUV-infection.

The output of the screen is broadly concordant with existing literature. A substantial fraction of validated antiviral compounds fall into functional classes that have been implicated previously in the replication of diverse RNA viruses, supporting the biological relevance of the screening platform. At the same time, the scale of the dataset and its validation across two distinct human cell types, A549 and HUVECs, provide insights into host dependencies difficult to achieve in hypothesis-driven studies.

Among the most robust findings is the enrichment of compounds targeting nucleotide biosynthesis, particularly inhibitors of inosine monophosphate dehydrogenase (IMPDH). IMPDH inhibitors such as mycophenolic acid and its derivatives have been reported to exhibit broad antiviral activity in vitro, including against hantaviruses in independent screens^12,19^. Our identification of multiple IMPDH inhibitors that suppress PUUV-infection in both A549 cells and HUVECs is therefore consistent with prior work and show that nucleotide availability is important for hantavirus replication.

Inhibitors of mTOR signalling constitute another recurrent class of antiviral compounds identified in this study. mTOR has been implicated in the life cycle of numerous RNA viruses (reviewed in^20^. The preferential efficacy of mTOR inhibitors in A549 cells compared with HUVECs adds an additional layer of context dependence. This observation suggests that the impact of mTOR modulation on PUUV infection may depend on baseline cellular state, or on how different cell types couple translational control to antiviral responses.

Several heat shock protein (HSP) inhibitors, particularly those targeting HSP90, also emerged among validated hits. The involvement of HSPs in viral replication is well documented across virus families, where they can facilitate protein folding, complex assembly, and stress adaptation^21^. The enrichment of HSP inhibitors in our dataset supports a role for proteostasis networks in hantavirus infection.

Unexpectedly, among the most effective and least toxic hits, we identified several beta-lactam antibiotics. While such compounds are not classically associated with antiviral effects, various classes of antibiotics have been shown to impair infection of diverse virus families, mostly through host-directed mechanisms^22^. To our knowledge, antiviral activity of beta-lactam antibiotics has not been reported previously. Given the reproducibility of our findings and the identification of multiple compounds from this class, this observation warrants further investigation.

Besides antiviral compounds, we identified 21 compounds with proviral effects in A549 cells among which epigenetic modifiers, particularly histone deacetylases inhibitors, constituted the most enriched cluster. Given their mechanism of action, HDAC inhibitors have a broad effect on the host’s transcriptional program. Addressing the molecular details behind HDAC-inhibitor mediated proviral effects on PUUV infection is necessary to establish a role for HDAC in restricting hantavirus replication or infection. One possible explanation is the reported role of HDACs in promoting cell’s immune response^23,24^.

The proviral effect could only be verified in A549 cells as the higher infection rate in HUVECs compared to A549 cells (~ 80% vs. ~20%), made it difficult to analyze pro-viral effects in HUVECs. Further studies are therefore needed to establish if the candidate provirals have cell type-specific or general effects.

In summary, the results from our drug repurposing screen are largely concordant with existing knowledge on host-directed antiviral strategies in the hantavirus field. In addition, our work has identified novel host dependencies during hantavirus infection that remain largely unexplored. As such, it provides a reference framework for prioritising host pathways for further mechanistic studies and for exploring new therapeutic avenues.

## Materials and methods

### Cell lines

Human lung epithelial A549 cells (ATCC CLL-185) were grown in minimal essential medium (MEM) supplemented with 7.5% fetal bovine serum (FBS), 100 U/ml penicillin, and 100 µg/ml streptomycin. Primary human umbilical vein endothelial cells (HUVECs) (Lonza, C2517A) were grown in endothelial cell medium (ScienCell, 1001) supplemented with 5% FBS, 1% endothelial cell growth supplement (ScienCell, 1052), and 1% penicillin/streptomycin solution. Cells were incubated at 37 °C and with 5% CO_2_.

### Puumala virus

PUUV (strain CG1820), sourced from Richars Elliott’s group in Glasgow, Scotland, was propagated in Vero E6 cells and titrated using end-point dilution assay as described earlier^25^. All work was done in BSL-2 laboratory, with all risk assessment protocols in place in accordance with institutional requirements.

In a standard infection protocol, cells were seeded and grown overnight. Medium was removed and Hank’s Balanced Salt Solution (HBSS), supplemented with 2% fetal calf serum, 1% penicillin-streptomycin and the desired amount of virus was added to cells. After 1-hour incubation at 37 °C, virus solution was removed, cells washed once with PBS, and fresh complete growth medium was added. This protocol was subjected to simplification, as described in the text.

### Screening

For the primary screen, A549 cells were seeded onto 384-well plates (Falcon 353962) that had been pre-spotted with the Drug Repurposing Hub library. Final concentration of library compounds was 10 µM (in triplicates); DMSO was used as a negative control.

For the validation screen, A549 cells or HUVECs were seeded onto 384-well-plates pre-spotted with the hits from the primary screen (150 antiviral and 25 proviral). Final concentrations of these compounds were 0.83 µM, 2.5 µM and 10 µM (in triplicates); DMSO was used as a negative control. After 6 hours incubation of cells and compounds, PUUV was added to the wells at MOI of 3. After a further 24-hour incubation, cells were fixed and directly stained for microscopy, as described below. Z-prime factor was calculated using formula: Z’ = 1-((3*σ_uninf_. + 3*σ_NegC)_/(|µ_uninf_ - µ_NegC_|)), where σ is the standard deviation and µ is the average. Signal-to-noise (S/N) ratio was calculated using the formula S/N = (µ_(uninf)_ - µ_(NegC)_)/σ_(NegC)_.

### Immunofluorescence microscopy

Cells were fixed with 4% formaldehyde for 15 min at room temperature, followed by 10-min permeabilization with 0.1% Triton X-100/PBS, 30 min blocking with blocking buffer (3% BSA/0.1% Tween-20/PBS), overnight incubation with primary antibody (convalescent patient sera; 1:1000) at 4 °C, and a 1-h incubation with secondary antibody (Invitrogen, A-21445) solution supplemented with 2 µM Hoechst 33342 (Thermo Scientific, 62249) and 1 µM CellTracker Orange CMTMR Dye (Thermo Scientific, C2927). PBS washes were done between each step. In the final screening assay, permeabilisation and blocking steps were ommitted.

Cells were then imaged using an IN Cell Analyzer 2200 (GE Healthcare) high-throughput microscope. Image analysis was performed using CellProfiler^15^ and data processing was done using the KNIME Analytics Platform.

### Image analysis and infection rate calculation

Image analysis was done in CellProfiler. Briefly, nuclei were segmented based on Hoechst 33342 signal, followed by cell body segmentation using CellTracker signal. Images with anti-Hantavirus signal were thresholded to generate a binary signal mask, which was then overlaid on segmented cells to identify infected cells. Both total and infected cell count was recorded. Infection rate was calculated as a ratio between the number of infected cells to total cell count. Infection rate and viability (i.e. total cell count) were normalised to the average values obtained for vehicle (DMSO)-treated samples.

### Statistical analysis

Statistical analysis was done in GraphPad Prism software using ANOVA with Dunnett’s test for multiple comparisons.

## Supplementary Information

Below is the link to the electronic supplementary material.

Supplementary Material 1

Supplementary Material 2

## Data Availability

All screening data and library metadata are available in the Supplementary material.
