# Supplementary material for: A drug repurposing screen identifies antiviral compounds against Puumala Orthohantavirus
Source: Sci Rep. 2026 Jun 25;16:19596. doi: 10.1038/s41598-026-57843-1 (PMC13303921; doi:10.1038/s41598-026-57843-1)
Supplement: Supplementary file 2 — Supplementary Material 2 [file 41598_2026_57843_MOESM2_ESM.pdf]

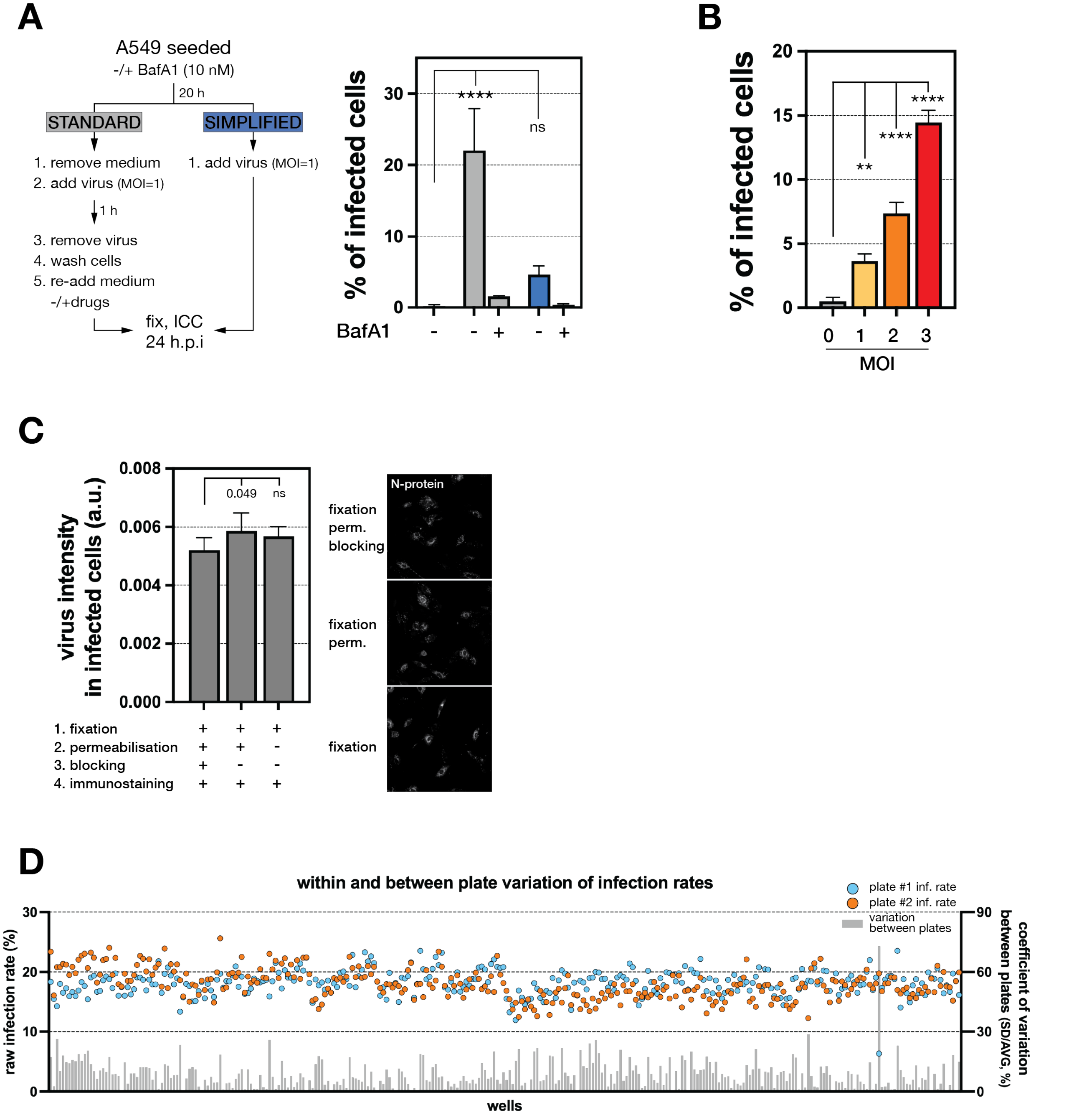

Figure S1. Simplification and optimisation of the screening assay.

A. Comparison of standard and simplified infection protocols. In the standard infection protocol, growth medium was removed, virus solution (without drugs) was added for 1 h at MOI=1, then removed, cells were washed, and fresh medium with or without compounds was added. In the simplified infection protocol, virus was added directly to the growth medium at MOI=1, without

removal and washing steps. Bar graph shows quantification of the infection rate. Bars indicate mean±SD (N=3); colours correspond to the infection protocol. Statistical analysis was done using ANOVA with Dunnett's test for multiple comparisons; ****, $p<0.0001$; ns, non-significant.

B. Optimisation of multiplicity of infection (MOI). A549 cells were infected with increasing MOI (0–3) using the simplified infection protocol. Bar graph shows the raw infection rate. Each bar indicates mean±SD (N=3). Statistical analysis was done using ANOVA with Dunnett's test for multiple comparisons; **, $p<0.01$; ****, $p<0.0001$.

C. Simplification of the immunostaining protocol. Bar graph (left) shows quantification of the virus signal in infected cells following different immunostaining protocols. Representative images are shown on the right. Each bar indicates mean±SD (N=3); a.u., arbitrary unit. Statistical analysis was done using ANOVA with Dunnett's test for multiple comparisons; ns, non-significant.

D. Within-plate and plate-to-plate variation of infection rate. Coloured circles represent infection rates (plotted on the left Y-axis) in each well (excluding outer wells) from two 384-well plates. Grey bars represent each well's coefficient of variation between the 2 plates (plotted on the right Y-axis).

FigS2

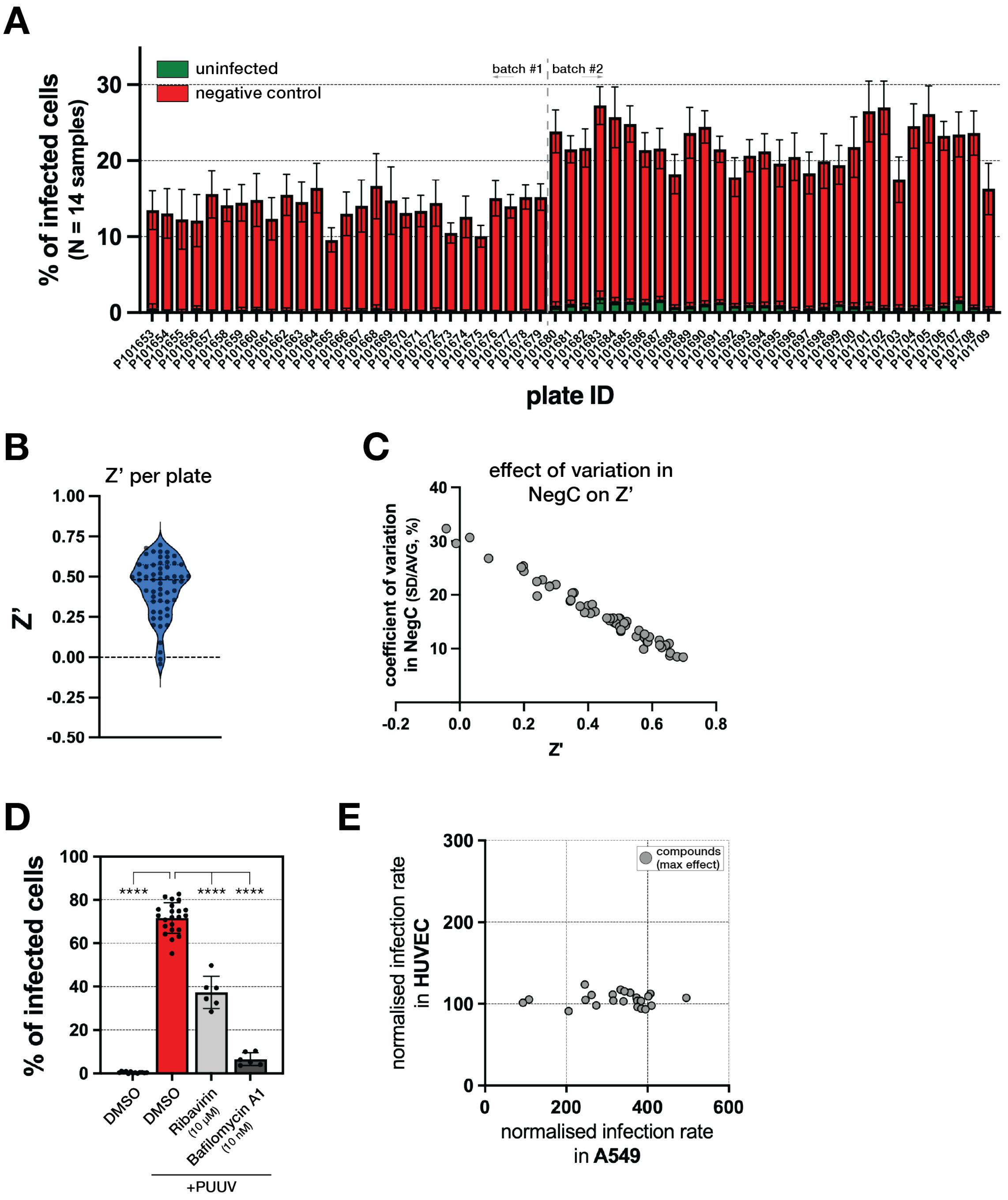

Figure S2. Primary screening quality control metrics.

A. Bar graph showing raw infection rate in uninfected (green) and infected vehicle-treated (red) samples. Each bar indicates mean±SD (n=14) in one screening plate. Dashed vertical line demarcates screening batches.

B. Violin plot showing the distribution of Z’ values calculated for each plate (dot) based on infected (negative control) and uninfected (positive control) samples. Mean Z’ for the entire screen was 0.41.

C. Scatter plot showing correlation between coefficient of variation in the negative control samples and Z’ factor. Each datapoint represents one screening plate.

D. Quantification of PUUV infection in HUVECs following treatment with vehicle (DMSO), 10 µM Ribavirin, or 10 nM Bafilomycin A1. Infection rate was calculated as the percentage of virus-positive cells. Dots represent individual wells, each with 2000–2500 cells. Bars indicate mean±SD. Statistical analysis was done using ANOVA with Dunnett’s test for multiple comparisons; ****, $p<0.0001$.

E. Comparison of maximal proviral effect between A549 cells and HUVECs. Each dot represents one compound; infection rates correspond to the concentration with the strongest observed effect.

FigS3

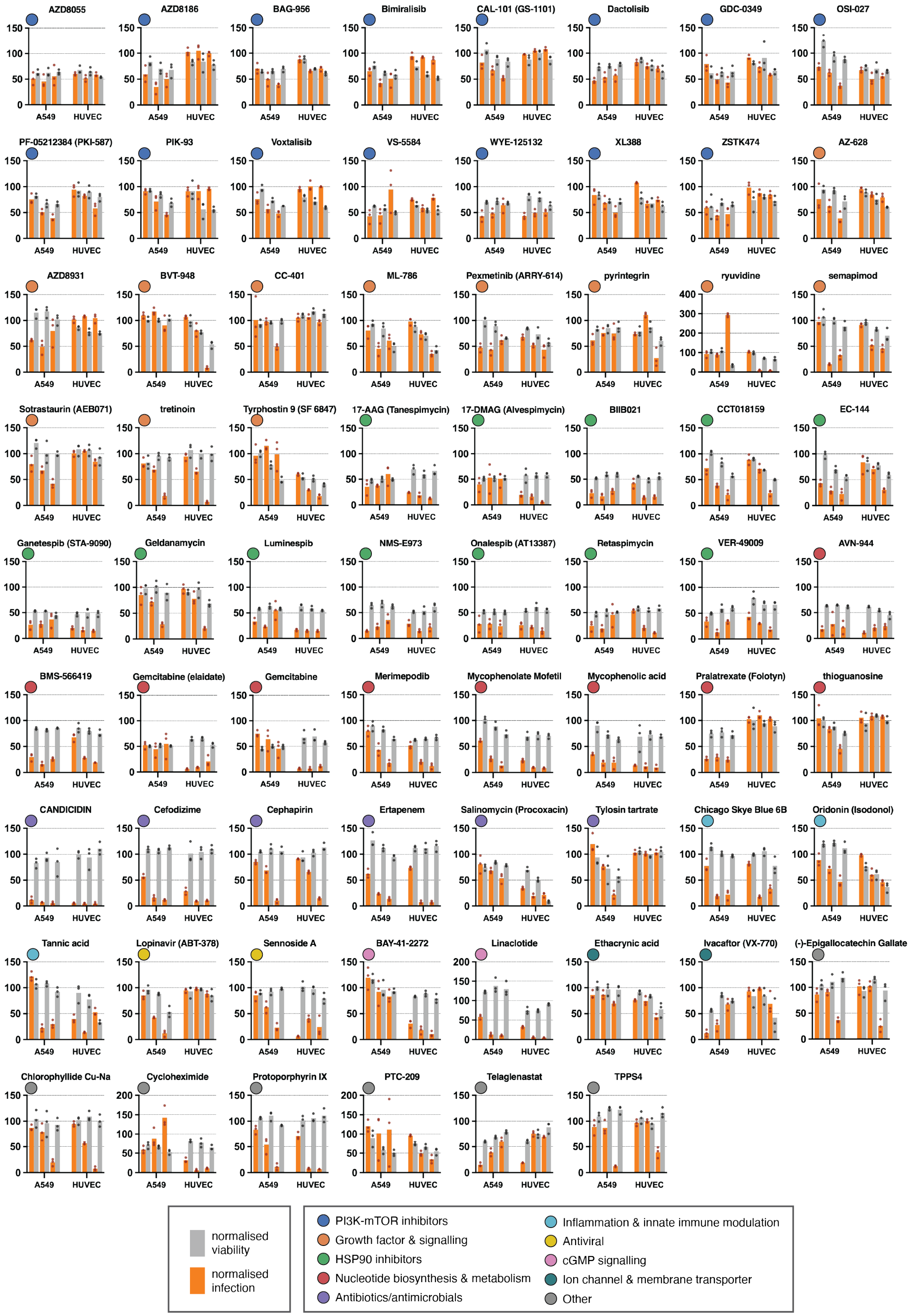

AZD8055
AZD8186
BAG-956
Bimiralisib
CAL-101 (GS-1101)
Dactolisib
GDC-0349
OSI-027
PF-05212384 (PKI-587)
PIK-93
Voxtalisib
VS-5584
WYE-125132
XL388
ZSTK474
AZ-628
AZD8931
BVT-948
CC-401
ML-786
Pexmetinib (ARRY-614)
pyrintegrin
ryuvidine
semapimod
Sotrastaurin (AEB071)
tretinoin
Tyrphostin 9 (SF 6847)
17-AAG (Tanespimycin)
17-DMAG (Alvespimycin)
BIIB021
CCT018159
EC-144
Ganetespib (STA-9090)
Geldanamycin
Luminespib
NMS-E973
Onalespib (AT13387)
Retaspimycin
VER-49009
AVN-944
BMS-566419
Gemcitabine (elaidate)
Gemcitabine
Merimepodib
Mycophenolate Mofetil
Mycophenolic acid
Pralatrexate (Folotyn)
thioguanosine
CANDICIDIN
Cefodizime
Cephapirin
Ertapenem
Salinomycin (Procoxacin)
Tylosin tartrate
Chicago Skye Blue 6B
Oridonin (Isodonol)
Tannic acid
Lopinavir (ABT-378)
Sennoside A
BAY-41-2272
Linaclotide
Ethacrynic acid
Ivacaftor (VX-770)
(-)-Epigallocatechin Gallate
Chlorophyllide Cu-Na
Cycloheximide
Protoporphyrin IX
PTC-209
Telaglenastat
TPPS4
A549
HUVEC
normalised viability
normalised infection
PI3K-mTOR inhibitors
Growth factor & signalling
HSP90 inhibitors
Nucleotide biosynthesis & metabolism
Antibiotics/antimicrobials
Inflammation & innate immune modulation
Antiviral
cGMP signalling
Ion channel & membrane transporter
Other

Figure S3. Dose response curves of validated antivirals.

A. Dose response profiles for all validated antiviral compounds. Normalised infection rate (orange) and normalised viability (grey) are shown for A549 cells and HUVECs. Bars represent mean of three replicates (dots). Coloured circles correspond to functional classes.

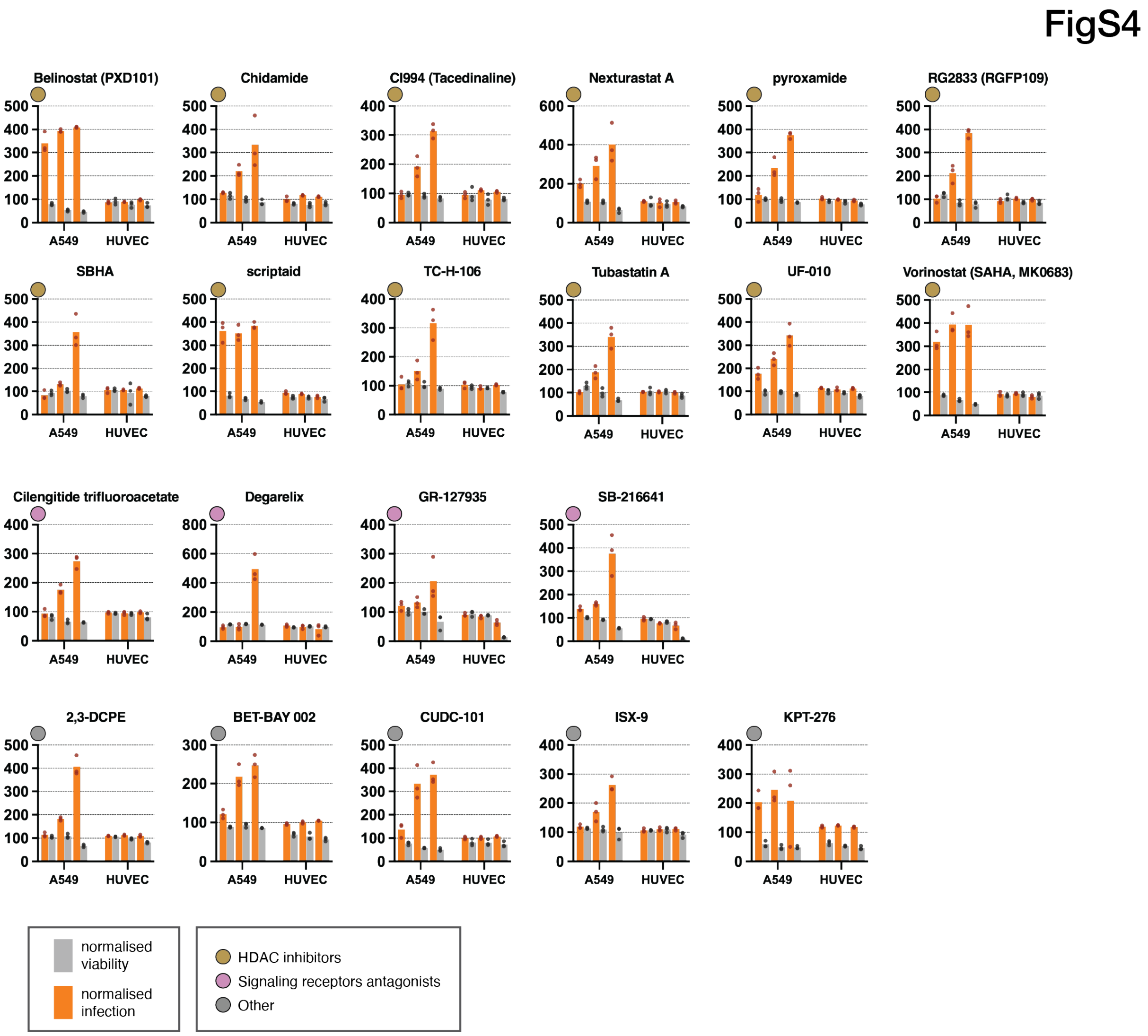

Figure S4. Dose response curves of validated provirals.

A. Dose response profiles for all validated proviral compounds. Normalised infection rate (orange) and normalised viability (grey) are shown for A549 cells and HUVECs. Bars represent mean of three replicates (dots). Coloured circles correspond to functional classes.
